# Some Conclusions as to the Effect upon Offspring of Marriages between Cousins-German

**Published:** 1898-09

**Authors:** John Inglis

**Affiliations:** Chicago


### SOME CONCLUSIONS AS TO THE EFFECT UPON OFFSPRING OF MARRIAGES BETWEEN COUSINS-GERMAN.

BY JOHN INGLIS, A.M., M.D., CHICAGO.

1. In the marriage of cousins-german the effect upon offspring will follow the law of heredity, the consanguinity *per se* being *nil*

2. Given high physical, moral, and intellectual development, the effect of the union would be beneficial rather than otherwise.

3. Special talents, either for good or evil, may be developed by intermarriage. This was the case in the historic musical family, the Bachs, where the musical talent was developed to a high degree of excellence.

4. In an effort to compare one hundred cases of marriages between cousins-german with one hundred average marriages where no relation existed, he took by lot from a physician's case-book, who had practiced in a town of fifteen hundred inhabitants for thirty years and knew their family histories well, the names of one hundred families and had this physician give him the record of these one hundred marriages with regard to sterility, pulmonary, mental, and congenital diseases. These were then compared with the marriages of cousins. The latter showed a lower percentage of sterile marriages and a slightly lower percentage of mental diseases. In pulmonary and congenital diseases there was about the same percentage of difference in favor of the former. In all other particulars the difference amounted to as little as any such comparisons can.

In the one hundred cases of those not related, 17 per cent. were sterile; in the cousins-german 14½ per cent. These figures agree very nearly with Huth's investigations.

5. The objection to cousins marrying, so far as there is an objection, is on the ground that very few families to-day are free from some tendency to weakness along some particular line. And it is equally true of these and of all marriages that heredity takes the line of least resistance.

A marriage between two healthy cousins coming of a line free from any hereditary defect is perhaps as fair as the average marriage.

6. But only carefully selected cases of first cousins should marry, and in no case where any hereditary moral or physical defect exists.

7. The early objections to such marriages were on religious, not scientific, grounds. Notwithstanding this, it is a significant fact that the Mosaic law permitted them.

These conclusions are in keeping with the most recent investigations of this subject, such as the younger Darwin and Huth of England, and Holbrook of New York in stirpiculture. —*Columbus Medical Journal.*

